# Impact of Plant Developmental Stage on Photosynthetic Acclimation to Elevated [CO_2_] in Durum Wheat

**DOI:** 10.3390/plants14142224

**Published:** 2025-07-18

**Authors:** Fernando Torralbo, Sergi Munné-Bosch, Carmen González-Murua, Iker Aranjuelo

**Affiliations:** 1 Department of Botany, Ecology and Plant Physiology, University of Cordoba, 14071 Córdoba, Spain; 2 Department of Evolutionary Biology, Ecology and Environmental Sciences, Faculty of Biology, University of Barcelona, 08028 Barcelona, Spain; smunne@ub.edu; 3 Department of Plant Biology and Ecology, University of the Basque Country (UPV/EHU), Apdo. 644, 48080 Bilbao, Spain; carmen.gmurua@ehu.eus; 4 Instituto de Agrobiotecnología (IdAB), Consejo Superior de Investigaciones Científicas-Gobierno de Navarra, 31006 Mutilva, Spain

**Keywords:** elevated [CO_2_], photosynthetic acclimation, phytohormones, carbohydrates, nitrogen

## Abstract

The response of plants to elevated atmospheric [CO_2_] is highly dynamic and influenced by developmental stage, yet its role in photosynthetic acclimation remains underexplored. This study examines the physiological and molecular responses of wheat (*Triticum durum*, var. Amilcar) to elevated [CO_2_] (700 ppm vs. 400 ppm) at two distinct developmental stages: the vegetative stage at the end of the elongation stage and the reproductive stage at the beginning of ear emergence (Z39 and Z51, respectively). Wheat plants at the developmental stage Z39, cultivated under elevated [CO_2_], maintained photosynthetic rates despite a carbohydrate build-up. However, at Z51, photosynthetic acclimation became more evident as the decline in Rubisco carboxylation capacity (Vc_max_) persisted, but also stomatal conductance and diffusion were decreased. This was accompanied by the up-regulation of the *CA1* and *CA2* genes, likely as a compensatory mechanism to maintain CO_2_ supply. Additionally, hormonal adjustments under elevated [CO_2_], including increased auxin and bioactive cytokinins (zeatin and isopentenyl adenine), may have contributed to delayed senescence and nitrogen remobilization, sustaining carbon assimilation despite biochemical constraints. These findings highlight the developmental regulation of photosynthetic acclimation, emphasizing the need for the stage-specific assessments of crop responses to future atmospheric conditions.

## 1. Introduction

Photosynthetic rate, and consequently plant growth, is influenced by atmospheric carbon dioxide concentration ([CO_2_]). Atmospheric CO_2_ levels are expected to exceed 900 µmol mol^−1^ by the beginning of the 22nd century [1], which is expected to enhance photosynthesis, potentially stimulating plant growth [2,3]. The initial stimulation of net photosynthetic rates may be retained or decreased under long periods of exposure to high concentrations of CO_2_ [4]. Changes in the photosynthetic efficiency of plants exposed to increased atmospheric [CO_2_] are linked to both stomatal and non-stomatal limitations. In addition, the physiological adaptation of leaves to elevated [CO_2_] is necessary to balance CO_2_ and water exchange through the leaf epidermis [5]. Elevated [CO_2_] usually reduces stomatal density and stomatal conductance (g_s_), which might have dramatic consequences on net photosynthetic rates. Concomitant with a reduction in stomatal conductance, prolonged exposure to elevated [CO_2_] also has an impact on photosynthetic electron transport [6,7] and the increase in non-structural carbohydrate contents [8,9]. Moreover, Rubisco carboxylation activity is highly affected by increased atmospheric [CO_2_] in plants with a C3 metabolism and thus, photosynthetic potential is ultimately affected [10].

The leaf’s non-structural carbohydrate contents are conditioned by the sink strength and the metabolic strength of the leaf. Moreover, the excess of non-structural carbohydrate contents in leaves affects photosynthetic performance, plant growth, and the ability to develop new sinks or expand the existing ones [11]. The carbon (C) imbalance between sink and source that occurs when the carbon assimilated in source leaves exceeds the requirements of sinks, limits photosynthetic capacity, and results in photosynthetic down-regulation in plants exposed long-term to increased atmospheric [CO_2_] [4]. Therefore, the synthesis and content of fructans, sucrose, and starch in leaves are highly regulated to control C fluxes and avoid photosynthetic inhibition. In addition, starch may participate in regulating sucrose metabolism, contributing to reduced shoot sucrose accumulation [11,12]. Carbon and nitrogen (N) metabolism are strongly interlinked, and the photosynthetic down-regulation mediated by carbohydrate build-up is more severe in N-deficient plants under high atmospheric [CO_2_] levels [13,14]. Nitrogen uptake and assimilation depend on the plant’s developmental stage, the N-form supplied, and the duration of exposure to enriched [CO_2_] environments [15,16]. Multiple molecules and mechanisms coordinate and regulate plant development. Among the large number of hormones, cytokinins are essential in modulating plant growth and development under enriched atmospheric [CO_2_] [17,18]. Cytokinins participate in many developmental and physiological processes like cell division, leaf senescence, chloroplast differentiation, chlorophyll synthesis, and nutrient assimilation [18]. Further, cytokinins improve the synthesis of chlorophyll precursors, which has a profound impact on photosynthetic assimilation [19,20]. Additionally, recent studies suggest that the amount of cytokinins plays a major role in the C assimilation and the N uptake of plants cultivated under elevated [CO_2_] conditions [16,21,22].

Wheat is one of the primary cereals produced and consumed globally and is cultivated across diverse regions, with yield and grain quality being highly conditioned by the environment [23]. Alongside growing climatic challenges is an increasing demand for cereals, which is expected to continue rising as the world’s population increases. Although the physiological responses to elevated [CO_2_] environments depend on many factors like the genetics and developmental stage of plants, the grain filling period in wheat plants is critical for grain yield and quality. During this period, the photoassimilates that sustain grain development are mobilized from various sources such as flag leaf photosynthesis, pre-anthesis assimilates, and ear photosynthates [24,25,26]. Consequently, the C-dynamics and nutrient sink-source activity undergo significant alterations. In addition, the transition between sink and source activities at different developmental stages demands a precise balance of C and N assimilation and remobilization processes. These are essential factors in determining the overall productivity and nutritional quality of wheat grains. However, only a few studies have assessed the impact of enriched [CO_2_] levels on plant physiology and yield across different phenological stages [27,28]. The current study emphasizes the molecular and physiological leaf adaptations involved in photosynthetic acclimation to elevated [CO_2_] at two distinct developmental stages (Z39 and Z51), integrating the role of cytokinin in maintaining photosynthetically active leaves over an extended period, and thus allowing for a greater mobilization of resources. Plants of the durum wheat commercial variety, Amilcar (*Triticum durum* L. cv Amilcar), which has moderate to high adaptability to different environmental conditions, were cultivated with nitric fertilization under two different atmospheric [CO_2_] levels (400 vs. 700 ppm). To help understand the reasons for photosynthetic acclimatation to elevated [CO_2_], we examined leaf gas exchange and non-structural carbohydrate and phytohormone contents, as well as the gene expression of carbonic anhydrases, ferredoxins, and nitrogen transporters in wheat plants at the Z39 and Z51 developmental stages [29]. We observed that the developmental stage conditions the photosynthetic responses of wheat to increased atmospheric [CO_2_].

## 2. Results

### 2.1. Biomass Accumulation

Plant biomass was not affected by exposure to elevated [CO_2_] at either the Z39 or Z51 developmental stages, although aboveground biomass tended to increase by more than 20% in plants grown under elevated [CO_2_] compared with those grown under ambient [CO_2_] conditions (Table 1). At the beginning of ear emergence, aboveground biomass increased in durum wheat plants by more than 70% under both [CO_2_] environments compared to the previous developmental stage at Z39. Moreover, elevated [CO_2_] tended to reduce leaf N content (%) by about 18% compared to ambient [CO_2_] conditions, regardless of the developmental stage. The leaf C content (%) and C/N ratio were not significantly different, regardless of developmental stage or [CO_2_] environmental conditions (Table 1).

### 2.2. Leaf Carbon Assimilation and Related Genes

**Figure 1:**
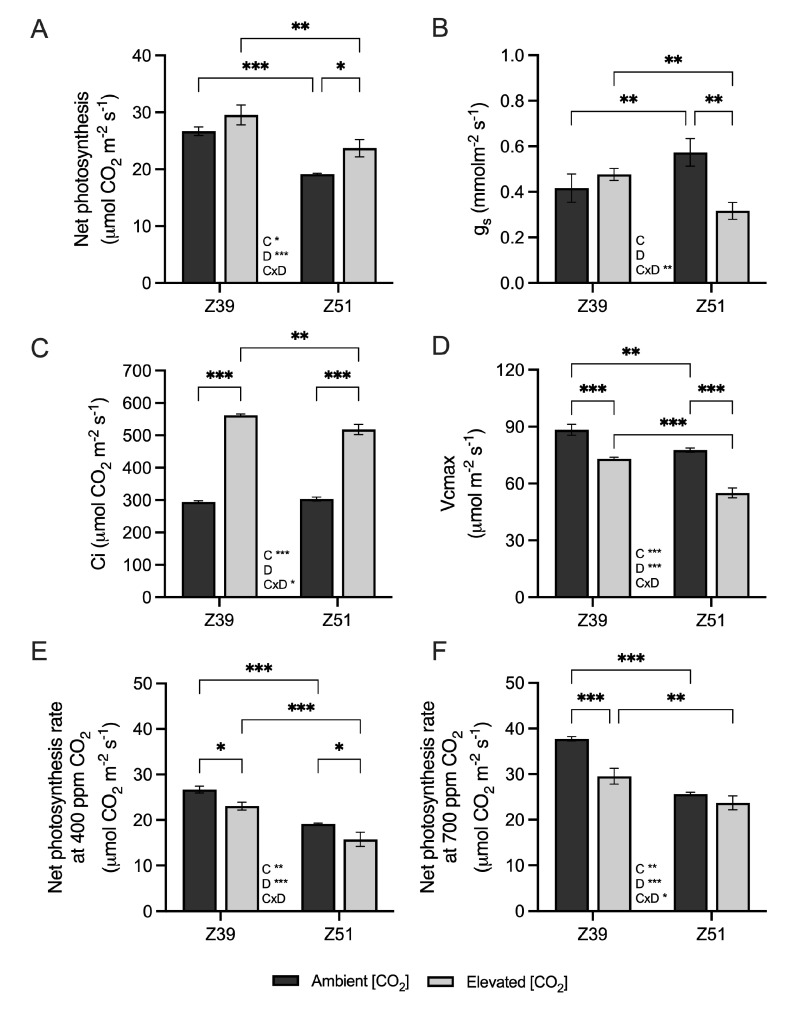
The effect of elevated CO_2_ (400 vs. 700 ppm) on (**A**) net photosynthesis (μmol CO_2_ m^−2^ s^−1^), (**B**) stomatal conductance (gs; mol CO_2_ m^−2^ s^−1^), (**C**) Rubisco maximum carboxylation rate (Vc_max_, μmol m^–2^ s^–1^), (**D**) intercellular [CO_2_] (Ci, μmol m^−2^ s^−1^) photosynthesis rates under reciprocal [CO_2_] conditions at (**E**) 400 ppm [CO_2_] (μmol CO_2_ m^−2^ s^−1^) and, (**F**) 700 ppm [CO_2_] (μmol CO_2_ m^−2^ s^−1^) in leaves of wheat fertilized with 15 mM of nitrate as the N source under ambient or elevated [CO_2_] levels (400 vs. 700 ppm, respectively) at two developmental stages (Z39 and Z51). Values are shown as the mean ± standard error (SE) of four biological replicates. Statistical analysis was performed by ANOVA (*p* < 0.05). Asterisks indicate significant differences (*p* < 0.05) between treatments according to Fisher’s LSD multiple comparisons test. Asterisks denote significance levels: * *p* < 0.05; ** *p* < 0.01; *** *p* < 0.001. [CO_2_], CO_2_ concentration; Dev., Developmental stage; [CO_2_]xDev., interaction of CO_2_ concentration and developmental stage.

At the end of the elongation stage, the wheat leaf photosynthetic rate and stomatal conductance were unaffected by elevated [CO_2_] (Figure 1A,B), whereas Ci increased as a consequence of higher [CO_2_] (Figure 1C). In addition, wheat plants exposed to enriched [CO_2_] conditions showed a depletion in leaf Vc_max_ compared to plants cultivated at 400 ppm [CO_2_] (Figure 1D). However, at the Z51 developmental stage, leaf photosynthetic rates were greater in plants exposed to increased atmospheric [CO_2_] than in plants grown at 400 ppm [CO_2_] (Figure 1A). Both g_s_ and Vc_max_ parameters in leaves of wheat plants grown under enriched [CO_2_] environments decreased compared to those grown at ambient [CO_2_] (Figure 1B,D). Overall, the leaf photosynthetic rate and Vc_max_ decreased with developmental stage regardless of the CO_2_ environment (Figure 1A,D), but only wheat plants at Z51 exposed to elevated [CO_2_] exhibited low intercellular [CO_2_] (Figure 1C). The leaf photosynthetic rate of plants grown under elevated [CO_2_] conditions measured at 400 μmol mol^−1^ decreased with respect to their growing conditions (Figure 1E). However, the leaf photosynthetic rate of plants grown under ambient [CO_2_], but determined at 700 μmol mol^−1^ of [CO_2_], increased by 41.5% at Z39 and 34% at Z51, which resulted in increases of 27.8% and 8.2% with respect to plants grown under elevated [CO_2_] conditions (Figure 1F).

**Figure 2:**
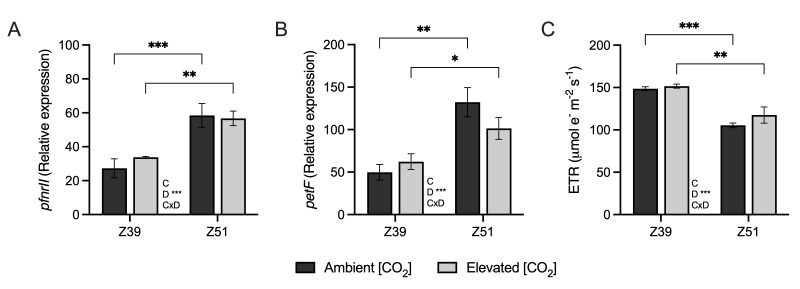
Effect of elevated CO_2_ (400 vs. 700 ppm) on relative gene expression of (**A**) *ferredoxin-NADP(H) oxidoreductase* and (**B**) *ferredoxin* and (**C**) and thylakoid electron transport rate (ETR, μmol e^–^ m^–2^ s^–1^) in leaves of wheat fertilized with 15 mM of nitrate as N source under ambient or elevated [CO_2_] levels (400 vs. 700 ppm, respectively) at two developmental stages (Z39 and Z51). Values are shown as mean ± standard error (SE) of four biological replicates. Statistical analysis was performed by ANOVA (*p* < 0.05). Asterisks indicate significant differences (*p* < 0.05) between treatments according to Fisher’s LSD multiple comparisons test. Asterisks denote significance levels: * *p* < 0.05; ** *p* < 0.01; *** *p* < 0.001. [CO_2_], CO_2_ concentration; Dev., Developmental stage; [CO_2_]xDev., interaction CO_2_ concentration and developmental stage.

**Figure 3:**
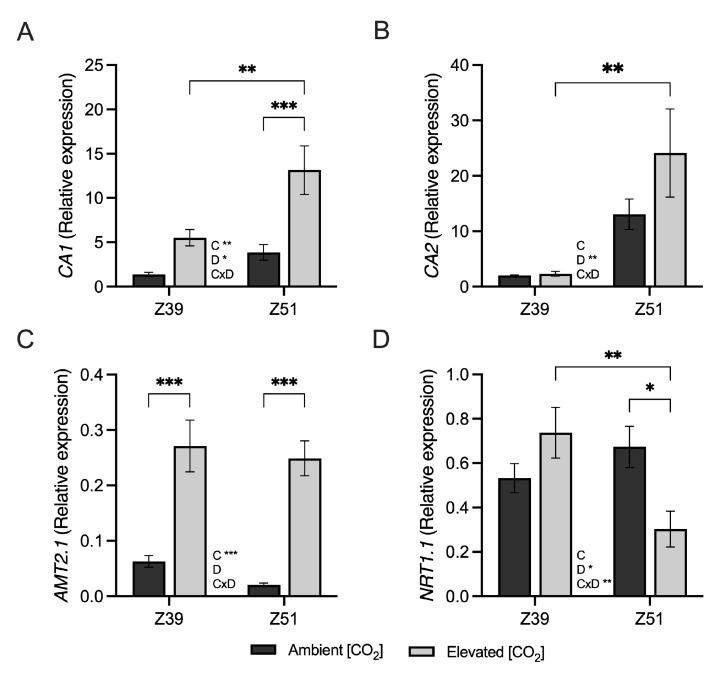
Effect of elevated CO_2_ (400 vs. 700 ppm) on relative gene expression of (**A**) *carbonic anhydrase 1*, (**B**) *carbonic anhydrase 2*, (**C**) ammonium transporter *AMT1.2,* and (**D**) nitrate transporter *NRT1.1* in leaves of wheat fertilized with 15 mM of nitrate as N source under ambient or elevated [CO_2_] levels (400 vs. 700 ppm, respectively) at two developmental stages (Z39 and Z51). Values are shown as mean ± standard error (SE) of four biological replicates. Statistical analysis was performed by ANOVA (*p* < 0.05). Asterisks indicate significant differences (*p* < 0.05) between treatments according to Fisher’s LSD multiple comparisons test. Asterisks denote significance levels: * *p* < 0.05; ** *p* < 0.01; *** *p* < 0.001. [CO_2_], CO_2_ concentration; Dev., Developmental stage; [CO_2_]xDev., interaction CO_2_ concentration and developmental stage.

The gene expression of *ferredoxin-NADP(H) oxidoreductase* and *ferredoxin* genes and the thylakoid electron transport rate (ETR) in leaves were unaffected by elevated [CO_2_], regardless of the developmental stage (Figure 2). Whereas the relative expression of *ferredoxin* and *ferredoxin-NADP(H) oxidoreductase* genes increased with developmental stage, the ETR decreased irrespective of the CO_2_ environment. Moreover, the relative expression of carbonic anhydrase genes increased alongside the developmental stage in wheat grown under elevated [CO_2_] (Figure 3A,B). The relative expression of the ammonium transporter *AMT2.1* gene was up-regulated by high-[CO_2_] conditions, regardless of the developmental stage (Figure 3C). In addition, genes related to the nitrate transporter NRT1.1 decreased in leaves of wheat plants at Z51 exposed to elevated [CO_2_] (Figure 3D).

### 2.3. Soluble Carbohydrate Accumulation

**Figure 4:**
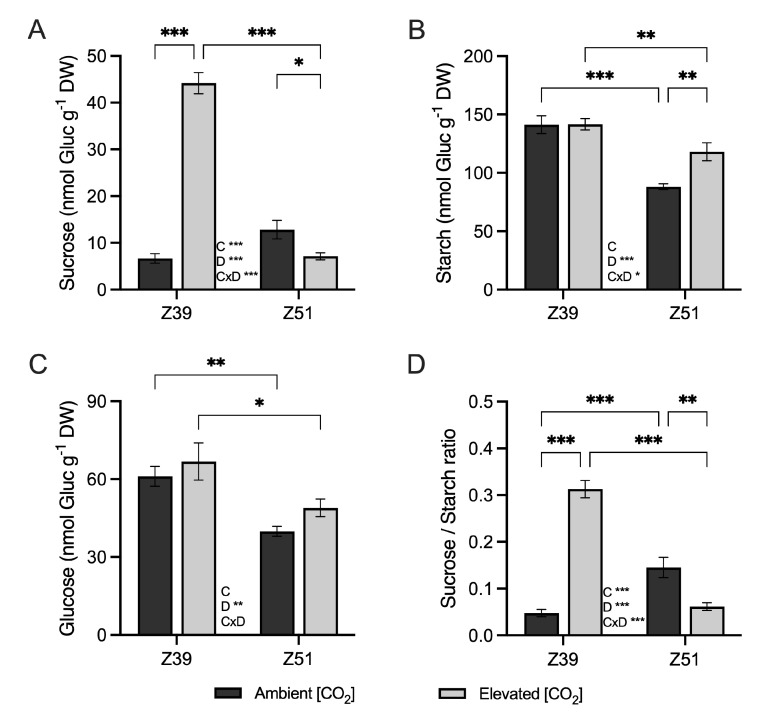
Effect of elevated CO_2_ (400 vs. 700 ppm) on (**A**) sucrose (nmol Gluc g^−1^ DW), (**B**) starch (nmol Gluc g^−1^ DW), (**C**) glucose (nmol Gluc g^−1^ DW) and (**D**) sucrose/starch ratio in leaves of wheat fertilized with 15 mM of nitrate as the N source under ambient and elevated [CO_2_] levels (400 vs. 700 ppm, respectively) at two developmental stages (Z39 and Z51). Values are shown as mean ± standard error (SE) of four biological replicates. Statistical analysis was performed by ANOVA (*p* < 0.05). Asterisks indicate significant differences (*p* < 0.05) between treatments according to Fisher’s LSD multiple comparisons test. Asterisks denote significance levels: * *p* < 0.05; ** *p* < 0.01; *** *p* < 0.001. [CO_2_], CO_2_ concentration; Dev., Developmental stage; [CO_2_]xDev., interaction CO_2_ concentration and developmental stage.

Leaf sucrose contents were highly influenced by exposure to elevated [CO_2_], plant developmental stage, and their interaction (Figure 4A). Sucrose contents in leaves of wheat at the end of elongation stage exposed to elevated [CO_2_] conditions were greater than in wheat grown under ambient [CO_2_] conditions, whereas the sucrose contents were depleted in leaves of wheat exposed to elevated [CO_2_] at the Z51 development stage. In addition, leaf starch contents decreased with developmental stage, regardless of the [CO_2_] conditions (Figure 4B). Although elevated [CO_2_] did not influence starch content in wheat leaves at the end of the elongation stage, the decline in starch content from Z39 to Z51 was more pronounced in plants grown under ambient [CO_2_] compared to those exposed to elevated [CO_2_]. Indeed, wheat plants under elevated [CO_2_] possessed higher starch contents at Z51 than wheat plants grown at ambient [CO_2_] (Figure 4B). Leaf glucose content was not affected by exposure to elevated [CO_2_], but the concentration of this carbohydrate diminished through development, irrespective of the [CO_2_] environment (Figure 4C). The sucrose/starch ratio, which indicates the synthesis and availability of sucrose for export from leaves relative to non-soluble starch, increased in leaves from Z39 to Z51 when plants were cultivated under 400 ppm [CO_2_], but under the elevated 700 ppm [CO_2_] conditions, the ratio decreased between the same developmental stages (Figure 4D).

### 2.4. Phytohormone Contents

**Figure 5:**
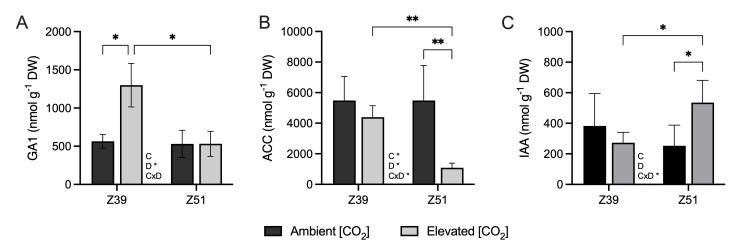
Effect of elevated CO_2_ (400 vs. 700 ppm) on (**A**) GA1 (nmol g^−1^ DW), (**B**) ACC (nmol g^−1^ DW) and (**C**) IAA (nmol g^−1^ DW) in leaves of Amilcar wheat plants (*Triticum durum*) grown with 15 mM of nitrate as the N source under ambient (dark bars) or elevated (gray bars) CO_2_ concentrations. Each value represents the mean ± SE of 4 biological replicates. Statistical analysis was performed by ANOVA (*p* < 0.05). Asterisks indicate significant differences (*p* < 0.05) between treatments according to Fisher’s LSD multiple comparisons test. Asterisks denote significance levels: * *p* < 0.05; ** *p* < 0.01. [CO_2_], CO_2_ concentration; Dev., Developmental stage; [CO_2_]xDev., interaction CO_2_ concentration and developmental stage.

Exposure to elevated [CO_2_] only promoted the accumulation of GA1 in leaves of wheat plants at the Z39 developmental stage (Figure 5A). On the other hand, the leaf content of the 1-aminocyclopropane-1-carboxylic acid (ACC), the precursor of ethylene, was significantly decreased in plants at Z51 grown under elevated [CO_2_] conditions (Figure 5B). Moreover, the indole-3-acetic acid (IAA) content was significantly greater in leaves of wheat plants at Z51 grown under elevated [CO_2_] conditions than in leaves of plants grown at ambient [CO_2_] conditions (Figure 5C).

**Figure 6:**
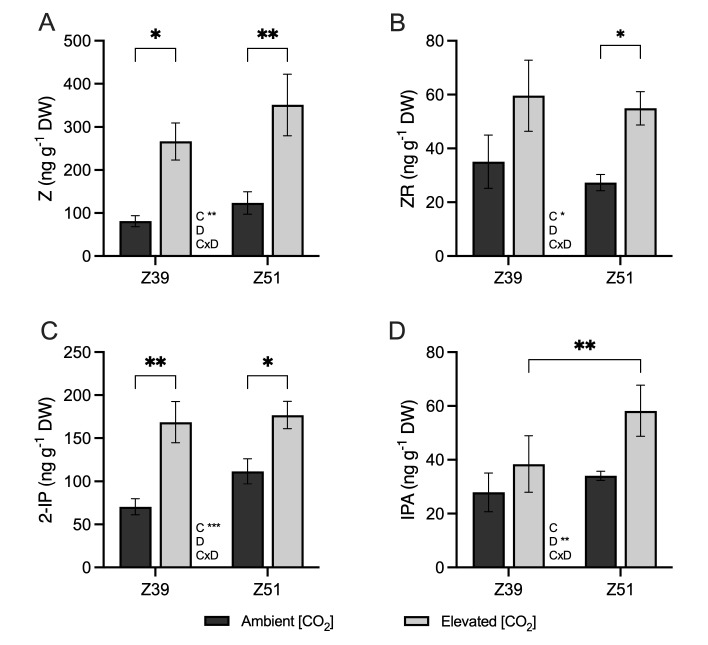
Effect of elevated CO_2_ (400 vs. 700 ppm) on (**A**) trans-zeatin (nmol Z g^−1^ DW), (**B**) trans-zeatin riboside (nmol ZR g^−1^ DW), (**C**) isopentenyl adenine (nmol 2-IP g^−1^ DW), and (**D**) endogenous isopentenyladenosine (nmol IPA g^−1^ DW) in leaves of Amilcar wheat plants (*Triticum durum*) grown with 15 mM of nitrate as N source under ambient (dark bars) or elevated (gray bars) CO_2_ concentrations. Each value represents mean ± SE of 4 biological replicates. Statistical analysis was performed by ANOVA (*p* < 0.05). Asterisks indicate significant differences (*p* < 0.05) between treatments according to Fisher’s LSD multiple comparisons test. Asterisks denote significance levels: * *p* < 0.05; ** *p* < 0.01; *** *p* < 0.001. [CO_2_], CO_2_ concentration; Dev., Developmental stage; [CO_2_]xDev., interaction CO_2_ concentration and developmental stage.

The leaf contents of trans-zeatin (Z), trans-zeatin riboside (ZR), and isopentenyl adenine (2iP) were not affected by developmental stage (Figure 6). Wheat plants exposed to elevated [CO_2_] exhibited higher Z content in leaves at both the Z39 and Z51 developmental stages than their respective controls under ambient [CO_2_] (Figure 6A). Although the leaf content of ZR at the end of the elongation stage was slightly higher in plants exposed to elevated [CO_2_] compared to plants at ambient [CO_2_], significant differences were only detected at Z51 (Figure 6B). On the other hand, wheat plants at Z51 grown under elevated [CO_2_] conditions showed significantly higher ZR content than plants at ambient [CO_2_]. The active cytokinin 2-IP content in the leaves of plants exposed to elevated [CO_2_] increased by 139% and 58% compared to their controls under ambient [CO_2_] at Z39 and Z51, respectively (Figure 6C), but leaf IPA contents were not significantly affected by [CO_2_] (Figure 6D).

## 3. Discussion

The effect of elevated [CO_2_] on photosynthetic capacity and biomass accumulation has been explored extensively in recent decades [2]. However, most studies have focused on a single developmental stage, overlooking the dynamic changes in plant physiology throughout phenological stages. As plants progress through their life cycle, shifts in carbon assimilation, resource allocation, and hormonal regulation shape their physiological responses to environmental factors, including elevated [CO_2_] [30]. To address this gap, the present work examines the physiological and molecular responses of wheat plants to elevated [CO_2_] at two distinct developmental stages—the end of the elongation phase (Z39) and the beginning of ear emergence (Z51)—providing a comprehensive view of how photosynthetic acclimation evolves over time.

Elevated [CO_2_] positively influenced net photosynthetic rates at Z51, but the concurrent reduction in stomatal conductance (gₛ) and maximum Rubisco carboxylation capacity (Vc_max_) suggested a progressive limitation of CO_2_ assimilation during photosynthesis. This was accompanied by a decrease in intercellular CO_2_ concentration (Cᵢ), likely driven by restricted CO_2_ diffusion and the biochemical down-regulation of photosynthesis [26,28,31,32]. Additionally, fluorescence measurements indicated a reduction in the ETR as plants transitioned from Z39 to Z51, suggesting the lower capacity for photochemical energy conversion under prolonged [CO_2_] exposure. The effect of elevated [CO_2_] on the maximum carboxylation rate has been previously aligned with an excess of carbon in the form of non-structural carbohydrates in leaves [26,28,32,33]. In the present study, wheat plants subjected to elevated [CO_2_] showed no significant differences in biomass compared to those grown under 400 ppm [CO_2_], despite increased Ci and carbohydrate accumulation. The accumulation of sucrose and starch displayed distinct patterns in flag leaves depending on the developmental stage, potentially due to increased assimilate demand in developing ears and grains, leading to a reallocation of resources from flag leaves [26]. According to previous studies, carbohydrate build-up in wheat grown under increased atmospheric [CO_2_] contributed to a high C:N ratio [34,35], indicating nitrogen dilution. Our findings confirm that elevated [CO_2_] tended to reduce leaf N content regardless of the developmental stage, which may be attributed to lower N demand and assimilation [36,37,38], enhanced CO_2_ assimilation [33], or the increased remobilization of N [28]. Although the photosynthetic acclimation to elevated [CO_2_] was evident, previous research suggests that an adequate N supply could mitigate the negative interaction between CO_2_ and N assimilation [35,39]. The depletion of leaf N content may be associated with lower demand for and the assimilation of N in these plants [37], an increase in CO_2_ assimilation [33], and enhanced N remobilization [28]. While acclimatory responses to elevated [CO_2_] were evident, del Pozo et al. [35] suggested that adequate N supply may mitigate the interaction between CO_2_ and N assimilation [39]. Additionally, in the companion study [40], we showed that the nitrate reductase and glutamine synthetase activities in wheat leaves exposed to elevated [CO_2_] were stimulated at the beginning of ear emergence (Z51), indicating that flag leaves were still contributing to the formation of new sinks. The down-regulation of Rubisco, combined with carbohydrate accumulation in upper leaves, suggests that photosynthetic down-regulation under elevated [CO_2_] in these plants was primarily driven by biochemical constraints [8,16,35]. Furthermore, the observed differential accumulation of non-structural carbohydrates reflects their role in sustaining new organ formation at distinct developmental stages.

Plants cultivated under elevated [CO_2_] environments often show a high C/N ratio in their leaves, suggesting N dilution in shoots [34] due to an imbalance in C sink/source [4]. Leaves of wheat exposed to elevated [CO_2_] possessed a higher C/N ratio, which agreed with the proposed imbalance between C and N metabolisms due to long-term exposure to elevated [CO_2_] [15,37]. In this regard, wheat at the beginning of ear emergence Z51 also displayed high gene expression of carbonic anhydrase, with a more pronounced increase in plants grown under elevated [CO_2_] conditions, as previously observed in wheat leaves [26,41]. The reduction in stomatal conductance associated with the up-regulation of *CA1* gene expression may be indicative of photosynthetic acclimation, with a subsequent depletion in the maximum carboxylate rate, as has also been shown in alfalfa [42]. The up-regulation of *CA* gene expression might also promote stomatal closure through the accumulation of higher bicarbonate content in guard cells [43], thus reducing the stomatal conductance observed at the beginning of ear emergence [26,27,41]. The lack of differences in *ferredoxin-NADP(H) oxidoreductase* and *ferredoxin* gene expression in leaf samples of wheat, irrespective of the [CO_2_], indicated that these plants had no limitations on reductants regarding the supply enough of NADPH for carbon fixation and N assimilation under elevated [CO_2_] conditions, which is in accordance with earlier studies in wheat [44,45]. Nevertheless, the up-regulation of *ferredoxin-NADP(H) oxidoreductase* and *ferredoxin* gene expression, observed at both ambient and elevated [CO_2_] conditions as advanced plant developmental stages, might indicate an intent of the plant to maintain the supply of reductant power caused by the decreased ETR.

Phytohormones are molecules that play a fundamental role in the response of plants to different environmental conditions [22]. Under elevated [CO_2_] conditions, leaves of wheat plants at the Z51 stage showed reduced ACC content and increased IAA content, which suggested a prolonged stay-green stage in these plants. The decrease in ACC, which is the precursor of ethylene, might be indicative of a delay in senescence and an extended photosynthetically active period [46]. Concurrently, the increase in leaf IAA content suggests the promotion of auxin signaling, which could enhance cell division and plant growth, improving sink strength to support the formation of the new sources [26,27]. Cytokinins influence almost all the developmental stages in plants, with roles including maintaining meristematic cells, forming chloroplasts and shoots, and delaying leaf senescence. Under 700 ppm [CO_2_], the increase in cytokinin content along with the increased IAA and reduced ACC leaf contents observed in plants at the Z51 stage indicate the tight hormonal adjustment required to support plant growth and development under elevated [CO_2_] conditions. An inverse relationship was previously described between endogenous cytokinin content and leaf senescence [47]. As mentioned before, the beginning of ear emergence leads to the remobilization of the majority of C and N stored in shoots [25], but a portion of the C is also assimilated by this new organ [26,48]. Additionally, cytokinins have a major effect at different levels in chloroplasts, promoting grana formation and increasing the contents of photosynthetic pigments and starch grains [49,50], but also reducing chloroplast senescence associated with auxins [51]. Here, we observed that wheat leaves grown under elevated [CO_2_] conditions exhibited higher contents of trans-zeatin, trans-zeatin riboside, and isopentenyl adenine than the leaves of plants cultivated at ambient [CO_2_]. This has been suggested to have a positive impact on increasing photosynthetic rates [47,51]. This increase in cytokinin levels due to the effect of elevated [CO_2_] has been previously demonstrated in cotton, Arabidopsis, and rice [21,52,53,54]. The elevated levels of auxin and cytokinin found in plants grown under enriched [CO_2_] environments suggest a prolonged stay-green period and delayed senescence in flag leaves, which might explain how wheat plants were able to maintain a prolonged metabolite allocation to new sources, despite the biochemical down-regulation of photosynthesis observed at this stage. In addition, high contents of cytokinin can also promote N distribution and transportation to shoots, influencing the expression of *AMT* genes [21,55], which is consistent with the enhanced expression of the *AMT2.1* gene observed in leaves of wheat grown under increased atmospheric [CO_2_]. Moreover, our results are aligned with previous results, suggesting that high a cytokinin content in rice plants under drought stress led to protection of photosynthetic processes and coordination between C and N assimilation of carbohydrates and amino acids, depending on plant requirements [56]. Furthermore, exogenous cytokinin exposure has been suggested to increase leaf N content in spring wheat plants exposed to elevated [CO_2_] [16], but also to increase the tillering and grain yield in winter wheat and spring barley [57]. Nevertheless, the large genotype variability in durum wheat, along with environmental factors such as water and nutrient availability, may lead to diverse physiological and molecular responses to elevated [CO_2_] [58]. Therefore, future studies should include a broader range of genotypes to better capture this variability.

## 4. Materials and Methods

### 4.1. Experimental Design and Plant Material

The seed germination of durum wheat (*Triticum durum* L. cv Amilcar) and growth conditions are described in Torralbo et al. [40]. Seed germination was synchronized by maintaining the seeds in trays with vermiculite–perlite (50:50%, *v*:*v*) and deionized water at 4 °C for 10 days in darkness. Each of the four biological replicates per treatment were grown in 5 L hydroponic pots with four wheat plants in two independent, fully controlled growth chambers (Phytotron Service, SGIker, UPV/EHU). The internal atmosphere of both growth chambers was set to [CO_2_] of 400 ppm and 700 ppm based on current atmospheric [CO_2_] and values simulated by 2100. Plants were grown under a day/night photoperiod of 14/10 h, a relative humidity of 50/60%, and an air temperature of 25 °C during the day and one of 17 °C at night. The intensity of light was adjusted to 550 µmol m^−2^ s^−1^. Nitrogen was applied at a rate of 10 mM of calcium nitrate in a modified Hoagland solution [59] that was replaced periodically. Physiological, biochemical, and molecular analyses were conducted on five-week-old wheat plants at the end of the elongation stage (Z39) and seven-week-old plants at the beginning of ear emergence (Z51) [29]. Four wheat plants per pot were harvested after leaf gas exchange analysis at each developmental stage and were combined for biomass determination. Leaf tissue samples were frozen in liquid N_2_ immediately after leaf gas exchange, pulverized using a mortar and pestle, and maintained at −80 °C until needed for biochemical and molecular analyses. The aboveground material was oven-dried at 80 °C for 72 h. The frozen leaves were lyophilized to determine carbon and nitrogen contents (%) and non-structural carbohydrates. For C and N contents, one milligram of lyophilized leaves was placed in small tin capsules and analyzed using a Flash 1112 Elemental Analyzer (Carbo Erba, Milan, Italy).

### 4.2. Leaf Gas Exchange and Chlorophyll Fluorescence Determinations

Leaf gas exchange was determined in fully expanded leaves using a Li-COR 6400XT portable photosynthesis system (LI-COR Inc., Lincoln, NE, USA). Net photosynthetic rate, stomatal conductance (g_s_) and intercellular CO_2_ (Ci) were measured under light-saturated conditions with a photon flux density (PPFD) of 1200 µmol m^−2^ s^−1^ at 25 °C and with the reference CO_2_ concentration of the respective growth chamber [40]. The maximum carboxylation velocity of Rubisco (Vc_max_) was estimated by the equation provided by Sharkey et al. [60] from the curves of net CO_2_ assimilation rate versus intercellular CO_2_ concentration (*A–Ci*) performed under saturated light conditions (1200 μmol m^−2^ s^−1^ PPFD) by decreasing the [CO_2_] from 400 to 100 ppm in three steps, followed by five steps to increase the [CO_2_] from 400 to 1800 ppm. The thylakoid electron transport rate (ETR) was measured using a Leaf Chamber Fluorometer (LFC 6400–40; Li-COR Inc., Lincoln, NE, USA) coupled to the Li-COR 6400XT portable photosynthesis system, 45 min after the dark period started.

### 4.3. Determination of Non-Structural Carbohydrates

Glucose, sucrose, and starch were extracted from ten milligrams of lyophilized leaf samples following the hydroalcoholic extraction method explained in Fuertes-Mendizábal et al. [61]. The soluble carbohydrates (glucose, fructose, and sucrose) were determined from the hydroalcoholic solution. Starch content was determined using a test kit (Boehringer Mannheim, Mannheim, Germany) on the dry pellet obtained from the hydroalcoholic extraction, following enzymatic digestion with α-amylase and amyloglucosidase.

### 4.4. Determination of Phytohormones

Gibberellic acid, 1-aminocyclopropane-1-carboxylic acid (ACC), indole-3-acetic acid (IAA), and the cytokinins trans-zeatin (Z) and trans-zeatin riboside (ZR), isopentenyl adenosine (IPA), and 2-isopentenyl adenine (2iP) were determined by ultrahigh-performance liquid chromatography tandem mass spectrometry (UHPLC-MS/MS) in multiple-reaction monitoring mode, according to Müller et al. [62]. For each of the four biological replicates, one hundred milligrams of powdered frozen leaf samples was homogenized with methanol–isopropanol–acetonitrile (50:49:1, *v*:*v*:*v*) for phytohormone extraction with two technical replicates. The extract was ultrasonicated for 30 min at 4 °C, followed by centrifugation at 14,000× *g* for 10 min at 4 °C to collect the supernatant. The extraction process was repeated until a colorless pellet was obtained. The pooled supernatants were passed through 0.22 µm PTFE filters (Phenomenex, Torrance, CA, USA), placed into HPLC vials, and injected into a UHPLC-MS/MS system for analysis. An Acquity UPLC System (Waters, Milford, MA, USA) coupled with an API 3000 triple quadrupole mass spectrometer (PE Sciex, Toronto, ON, Canada) was used for the analyses. For quantification, deuterium-labeled compounds were employed as internal standards.

### 4.5. Extraction of Leaf RNA and Relative Gene Expression

Total leaf RNA was extracted from leaf powdered samples using a Nucleospin RNA plant kit (Macherey-Nagel). The RNA integrity and quality were assessed using a 1.5% agarose gel. Up to 1 µg of RNA was reverse-transcribed using a PrimeScript RT reagent Kit (Takara Bio Inc., Saint-Germain-en-Laye, France). The expression of ferrodoxin-related genes (*pnfrII* and *petF*), carbonic anhydrases (*CA1* and *CA2*), and N transporters (*AMT2.1* and *NRT1.1*) was determined by quantitative reverse transcription PCR (RT-qPCR) in a StepOne Plus Real Time PCR System (Applied Biosystems). Each PRC reaction consisted of 200 nM of gene-specific primers [33], 2 µL of ten-times-diluted cDNA, and the SYBR Premix ExTaq (Takara Bio Inc.). The PCR thermal protocol consisted of treatment at 95 °C for 10 min, followed by 40 cycles (95 °C for 15 s and 60 °C for 1 min). The melting curve was included as a final step. Relative gene expression was measured with the ∆Cp method. RNase L inhibitor-like protein and actin genes were used as reference genes to normalize the expression [63].

### 4.6. Statistical Analysis

The normality and heterogeneity of variance across the treatments were analyzed using SPSS version 29 (Chicago, IL, USA) with the Shapiro–Wilk and Levene tests, respectively. To assess the effects of [CO_2_], developmental stage, and their interactions, two-way ANOVA with Fisher’s LSD multiple comparisons was performed using GraphPad Prism version 10.0.0 (Boston, MA, USA).

## 5. Conclusions

The current study demonstrates that durum wheat exhibits a differential response to elevated [CO_2_], depending on the developmental stage, with distinct physiological and biochemical adjustments. By analyzing responses at both the end of the elongation stage (Z39) and the beginning of ear emergence (Z51), we described the temporal pattern of photosynthetic down-regulation, primarily coordinated by a reduction in Vc_max_, carbohydrate accumulation, and nitrogen depletion. This study shows the importance of the compensatory up-regulation of carbonic anhydrase genes and the pivotal role of hormonal modulation. Specifically, it showed increased levels of cytokinins and indole-3-acetic acid (IAA) in mediating delayed senescence and extending the stay-green period under elevated [CO_2_] conditions. These findings highlight the mechanisms by which durum wheat maintains metabolite allocation to developing sinks, with the potential to ensure grain filling and yield stability in future climate scenarios. By integrating physiological, biochemical, and molecular analysis, our findings underscore the necessity of a comprehensive understanding of C and N metabolism, and they offer innovative targets for breeding climate-resilient wheat varieties to optimize productivity under future climate change scenarios, contributing to global food security.

## Figures and Tables

**Table 1:** Shoot dry biomass (g), leaf N and C content (%) and the C/N ratio in wheat fertilized with 15 mM nitrate as N source under ambient or elevated [CO_2_] levels (400 vs. 700 ppm, respectively) at two developmental stages (Z39 and Z51). Values are shown as the mean ± standard error (SE) of four biological replicates. Different letters denote significant differences (*p* < 0.05) between treatments by ANOVA using the LSD post hoc test. Significant differences are indicated by asterisks: * *p* < 0.05; *** *p* < 0.001. [CO_2_], CO_2_ concentration; Dev., developmental stage; [CO_2_]xDev., interaction of CO_2_ concentration and developmental stage.

Zadoks	[CO_2_]	Shoot Dry Biomass (g)		N Content (%)		C Content (%)		C/N Ratio	
Z39	400	5.28 ± 0.11	b	2.4 ± 0.26	ab	44 ± 0.37	a	18.97 ± 2.08	ab
	700	7.67 ± 0.51	b	1.85 ± 0.04	b	43.53 ± 0.47	a	23.62 ± 0.52	a
Z51	400	21.77 ± 2.73	a	2.69 ± 0.25	a	43.45 ± 0.47	a	16.68 ± 1.83	b
	700	26.26 ± 1.1	a	2.18 ± 0.17	ab	43.5 ± 0.49	a	20.28 ± 1.59	a
[CO_2_]: * Shoot Dry Biomass (g), * N content (%), * C/N ratio									
Dev.: *** Shoot Dry Biomass (g), * N content (%), * C/N ratio									

## Data Availability

Data of this study are available from the corresponding authors upon reasonable request.
